# Peer support after clinical incidents in veterinary care: Adopting the RISE (Resilience In Stressful Events) program

**DOI:** 10.1371/journal.pone.0341324

**Published:** 2026-01-27

**Authors:** Lisen Schortz, Liz Mossop, Catherine Oxtoby, Annika Bergström, Albert W. Wu

**Affiliations:** 1 AniCura, Stockholm, Sweden; 2 Shefﬁeld Hallam University, Shefﬁeld, United Kingdom; 3 Veterinary Defence Society, Knutsford, United Kingdom; 4 AWAKE Hospital, Stockholm, Sweden; 5 Armstrong Institute for Patient Safety and Quality, School of Medicine, Johns Hopkins University, Baltimore, United States of America; 6 Johns Hopkins Bloomberg School of Public Health, Johns Hopkins University, Baltimore, United States of America; Universiti Sains Malaysia - Kampus Kesihatan, MALAYSIA

## Abstract

Veterinary professionals often experience emotional distress after clinical incidents, affecting well-being and job performance. This study explores the adaptation and implementation of the Resilience In Stressful Events (RISE) program, originally developed for human healthcare, to support veterinary clinicians. RISE provides peer support through trained responders, focusing on emotional care rather than event details. Findings indicate that the RISE program is both acceptable and feasible for veterinary settings, with minor adaptations, such as including veterinary examples, to fit veterinary specific needs. The study highlights the importance of structured support systems in improving the mental health and retention of veterinary professionals, suggesting that programs like RISE could play a crucial role in enhancing clinician wellbeing and care quality.

## Introduction

Clinicians in both human and veterinary medicine may experience emotional distress following medical incidents that harm patients [1,2]. Such events can negatively affect job satisfaction and overall well-being [3–5], and may undermine clinical confidence [6], potentially compromising care quality and increasing the risk of further incidents and defensive practices [7–10]. Whitnall and Simmonds (2021) highlighted a widespread lack of support in clinical settings, with most participants reporting insufficient assistance. Peer support is a key factor in clinician well-being [11] and its absence can hinder coping and recovery after distressing events [1,5,12].

In veterinary care, Kogan et al. (2018) found that near misses (74%) and adverse events (30%) are common, often leading to reduced mental health and sleep quality. Recent studies show that patient safety events significantly impact veterinary professionals’ well-being and career intentions, with many expressing a strong need for peer-based support [13]. While the frequency of incidents is not well established, one small study reported five per 1,000 care visits [14] and a larger study found rates ranging from 0.5 to 16.7 per 1,000 [15]. A recent study on second victims in veterinary anesthesia revealed that over half of participants had considered leaving their jobs due to incident-related stress [16]. Veterinarians also face elevated risks of depression, anxiety, and suicidal ideation, often linked to work-related stress [17,18]. Irwin and colleagues (2023) demonstrated how incivility, such as disrespectful comments and lack of support, exacerbates these issues, affecting decision-making, collaboration, and care quality [19], and reinforcing the need for systemic support [5,20].

Support strategies for clinicians include professional counseling, peer support programs, and clinician-client conversations [21–23], all aimed at facilitating recovery and reducing fear of retribution. In human healthcare, several formal healthcare worker support programs (HWSPs) have been developed [24–27], typically offering timely, confidential peer support to reduce suffering and stigma [1,28]. One example is Resilience In Stressful Events (RISE), developed at Johns Hopkins Hospital [28]. RISE uses a peer support model with formal training for leaders and volunteer peer-responders, who provide 24-hour on-call support. Implementation involves two 8-hour training sessions, one for leaders to tailor the program to their organization, and one for responders to deliver support. Training includes lectures, videos, role-play, and a guidebook with implementation tools. Support focuses on emotional responses rather than event details and is delivered in person or by phone or internet. Responders debrief after each session to support their own well-being and gather feedback for program improvement [29,30].

Program usage varies across departments; for example, emergency clinicians in one pediatric organization accounted for 62% of all calls, indicating differing needs across services. Patient death is consistently the most common reason for seeking support [31,32].

Quantifying HWSP effectiveness is challenging due to limited empirical evidence [27,28,33,34]. To protect confidentiality, effectiveness is often assessed through responder perceptions. HWSPs have been linked to improved personal autonomy, reduced turnover [35], and enhanced institutional safety and support culture [34]. Burlison and colleagues (2017) developed a validated tool to measure program impact using absenteeism and turnover intentions [36], which has been successfully adapted and validated internationally [37,38].

Despite similar impacts of clinical incidents in veterinary care, evidence-based support interventions remain underexplored [5]. This study aimed to (i) adapt key aspects of the RISE program, including training content, delivery format, and responder roles, for veterinary settings; (ii) pilot the adapted program in a veterinary practice; and (iii) evaluate its feasibility and acceptability by assessing participant engagement, perceived usefulness, and practical implementation challenges.

## Methods

**Fig 1:**
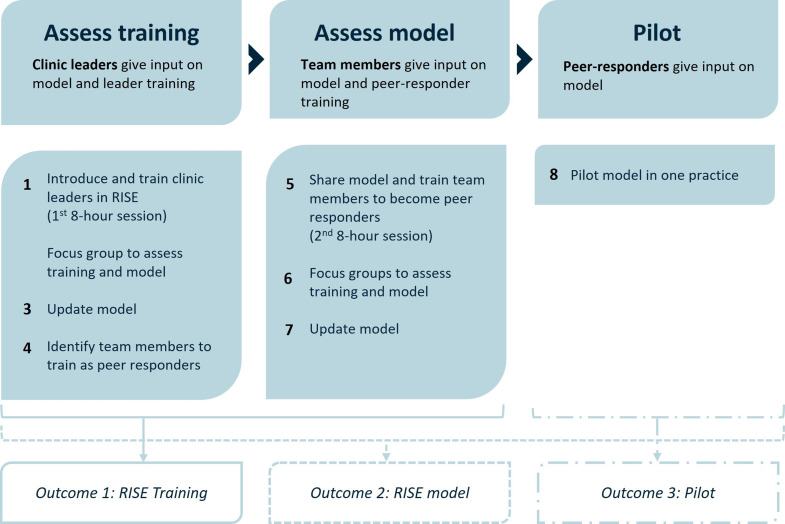
Overview of the study design including steps taken to adapt the RISE peer-support model developed for human healthcare for veterinary practitioners.

RISE was adapted using qualitative feedback from a group of veterinary clinicians undertaking implementation of the program. A two-stage approach was adopted, first involving clinic leaders and second in training peer-responders, adapting the model iteratively via focus group data at each stage (Fig 1). The final program was pilot tested in one practice. The RISE scheme, as a CSP, was chosen as part of a cooperation between the author’s company and Johns Hopkins.

Participants were recruited between March 15^th^ and May 16^th^ 2022 via word of mouth from a small animal veterinary group operating on mainland Europe, using a snowball sampling technique, which may introduce selection bias as participants are more likely to refer colleagues with similar perspectives or experiences. Participation was voluntary. The initial training was held online and divided into two four-hour long sessions. Following training, participating leaders identified potential peer-responders in their teams who were invited to participate.

This research is presented in accordance with the COREQ (COnsolidated criteria for Reporting Qualitative research) guidelines [39]. Several items were adapted for the online format: written consent was replaced by audio-recorded verbal consent, as approved by the University of Lincoln ethics committee, field notes and non-verbal observations were limited; and transcripts were not returned to participants for correction. Items relating to the physical setting and presence of non-participants were not applicable. Ethical approval was granted by the University of Lincoln ethics committee (2022–8861).

AniCura and Mars Veterinary Health provided logistical support and access to clinical sites for the implementation of the RISE program. However, neither organization was involved in the design of the study, data collection, analysis, or interpretation of the findings.

### Data collection and analysis

Clinical leaders took part in the RISE leadership training delivered by the Johns Hopkins training team, and a subsequent focus group. The focus groups were conducted in English or Swedish, by a lead researcher previously known to a minority of participants. During the focus group, questions were guided by those asked in the RISE debrief survey (Appendix 1). In the second phase, team members took part in the second RISE peer-responder training to become peer-responders. Following training, participants participated in a focus group and shared their thoughts and perspectives on the model and training. Focus Groups (FG) were recorded with participants informed about the study’s aims, confidentiality, and their right to withdraw at any time. The number of FGs was guided by the point when theoretical saturation was met. We defined saturation as the point at which there were sufficient data to provide a coherent analysis; not that further interviews might not yield new concepts. The recordings were transcribed and descriptive qualitative analysis was used to describe and summarize aspects of importance [40,41]. Quotes were translated from Swedish to English by LS and verified for correctness by AB.

Microsoft Excel (v. 2019 and 2021) was used to organize and manage the qualitative data. Coding and theme identification were led by LS, with marked passages and memos continuously discussed and reviewed with the wider research group to enhance validity and reduce individual coding bias. If there was a disagreement during coding or theme identification, the issue was discussed among the research team until consensus was reached. The outcome was used to evaluate the RISE training and inform the model.

### Pilot test and analysis

The preliminary veterinary model of RISE was pilot tested voluntarily, between June and October, in one Italian clinic, providing primary and referral care 24/7, with two peer-responders who supported 48 team members. Support could be given face-to-face or by phone between 9 AM and 5 PM, five days a week. Team members were encouraged by managers to seek support when in distress. Experience running and managing the program after five months was captured in two debriefing sessions with responders, alongside a debrief questionnaire filed after an encounter. Descriptive qualitative analysis was used to group and summarize areas of importance to answer the research questions [40,41]. Themes were identified by reading the transcripts, followed by marking sentences. The excerpts were grouped considering relevance and then refined to themes. The preliminary themes were complemented with quotes and verified with the raw data and wider research group for validity. The data captured in the debrief questionnaire was in English and descriptively summarized.

## Results

### Training and model

Two training sessions were conducted in May 2022 to support veterinary and healthcare professionals in leadership and peer-response roles. Nineteen female leaders from Sweden, Italy, the Netherlands and Spain, with both clinical and non-clinical backgrounds and responsibilities, participated in the first leadership RISE training session and the first focus group. Seventeen female participants, with both clinical and non-clinical backgrounds and responsibilities participated in the second RISE peer-responder training session. Five of these participated in both training sessions. Fourteen were Swedish and three were Italian. Seven were veterinarians, four were nurses and six held various degrees in human resources, business and finances. They had between eight and 30 years of experience, with an average of 14 years in the field. Several of the participants had a personal interest in behavior, self-care, wellness and or coaching.

### Phase 1

Five focus groups were held with three to seven participants in each. Emergent themes identified were ‘contextualising training’ and ‘differences in veterinary and cultural context’.

#### Contextualising training.

The RISE training was relevant and transferable to the veterinary profession. The only adaptation, to increase relatability, was to have examples and videos from the veterinary field instead of human healthcare. The simulation exercises were found especially helpful to understand how to provide support, when to do so and how it felt.

*“I found the exercise … very good, even though I am uneasy during role plays. Supporting something that didn’t need support… felt fake”* (FG3).

Most felt the program effectively built skills for running a pilot and training peers, though some suggested shortening it and practicing in informal settings before launching the pilot.

#### Differences in veterinary and cultural context.

Differences between veterinary and human healthcare were explored to adapt RISE for veterinary use. In veterinary care patients cannot speak, life-and-death decisions are common, and each visit involves cost for the client, affecting interactions and care paths. Veterinary care also tends to occur in smaller, multi-species spaces using “what is available,” unlike larger, purpose-built healthcare facilities. These differences led to suggestions for training content on the complexity of non-verbal patients and financial impacts on care and accountability. Participants also felt the training should be adapted for European contexts, with Swedish participants saying the U.S. developed material was “*too happy and lacked weight and seriousness*” (FG1).

Following the initial adaptation and feedback from clinic leaders in Phase 1, Phase 2 focused on training peer-responders and further refining the model based on their experiences.

### Phase 2

#### Role expectations.

Support needs vary, from immediate help to providing support for more chronic stresses. “I was working the night shift last night, and the clients coming are completely different... It will be challenging to provide support for all of that” (FG2). Clarifying the HWSP’s purpose was seen as essential. The peer-responder’s role was to help colleagues process experiences: “the responder is there to give support, not advice about what to do next or tell them what is wrong or right” (FG1). Participants noted it could be hard to resist problem-solving: “We see this type of situation every day... But what I am not used to, is not being able to do something about it...” (FG2). Training highlighted the value of listening rather than fixing the problem.

#### Peer-responder compensation: Balancing ethics and effort.

The most discussed topic was compensation models for responders. Ideas included hourly pay, on-call stipends, bonuses, engagement activities, or scheduling support during work hours. The original RISE model relies on unpaid volunteer peer-responders and some felt asking for money was ethically wrong: “I cannot see that there should be any large compensation for supporting a colleague” (FG4). While no consensus was reached, the preferred option was integrating on-call hours into regular work schedules.

#### Institutional resource provision.

Participants agreed that leadership support at both practice and organizational levels is essential for successful implementation. Time allocation and clear communication channels were seen as key barriers and enablers. Many noted past initiatives had failed due to shifting priorities: “It depends on what the flavour of the month is. [sigh] I would rather see us under-promise and over-deliver on this one” (FG4).

#### Importance of evaluating RISE.

*“I have a feeling this is a really good thing, but how do we know? We should at least measure it in some way during the pilot”* (FG1)*.* The absence of follow up with the clinician after an encounter was considered an issue. Participants believed that it would be advantageous for both the responder and the peer, but this was against the recommendation from the RISE trainers considering the inappropriateness of contacting a person in distress. That the responder had a debrief after an encounter to capture their perspective of effectiveness of the encounter was found vital to learn collectively.

#### Model design: Single or multi-site framework.

When participants reviewed the format of the RISE program it highlighted elements to consider when shaping a model to fit the organization at hand. For one, the RISE program was designed for Johns Hopkins single site location with 8,500 associates and 1,000 bed capacity. That was different from the company’s, with 450 locations, run by teams varying from five to 250 members. Therefore, two model frames were proposed: one single site, with local support in each location, and one multi-site, with one central team supporting all locations. Each model with different advantages: “*You want to talk to someone who is not at the same clinic”* (FG2). Independent of model, it consists of the same five elements as in RISE, with the only difference being with caller feedback, suggested to be tailored to what fits.

#### Activating support and coordinating response.

The preferred way to inform teams and activate support was via a QR code, “to scan a QR code is easy” (FG4). Coordination methods were discussed, but no clear preference emerged. The internal telecom system was lacking in confidentiality. A third-party platform offered a dedicated workspace, call/message handling, usage tracking, and scheduling tools, but came with a financial cost.

#### Internal marketing.

Participants agreed that clear resources and communication materials were needed to launch a HWSP (Table 1). *“To be more successful more awareness before. Need more time to involve more clinic. Maybe 4-5 months before. Enthusiastic about the program”* (FG3). Sharing new initiatives is challenging due to heavy workloads, shift work, and information overload.

**Table 1:** List of resources and communication materials needed to successfully launch a healthcare worker support program.

Resource	Purpose of document
Overview of what organizational support is available, when it should be used, and how to access it	• Allows team members to choose the best support options for their specific needs • Facilitates onboarding of new team members • Helps the organization identify and fill gaps in current support offerings • Enables the organization to stay up-to-date with research and trends in the field • Contributes to creating a positive work environment that supports employee well-being
When to ask for support and how to activate it	• Provide clarity and timely access to support
On-call schedule for responders*	• Organizes and structures the support program • Ensures responders are available at designated times • Prevents responders from being overburdened • Makes support available during periods of high stress or specific events
Debrief schedule for responders	• Organizes and makes the program accessible • Supports regular and effective assistance for responders
Training material for responders	• Provides standardized and consistent training • Ensures responders have the necessary knowledge and skills for their role
Information material for leaders	• Explains the program’s purpose and operation • Ensures leadership support

*only for multi-site model.

### Pilot test

Four themes were identified in the pilot: value of listening, challenges to focus exclusively on providing support, limited value of debrief form, and importance of marketing.

#### Value of listening.

Responders reported that RISE training helped them adopt a supportive approach in daily work, *“It is super important to listen!”*. They suggested all managers take the training to learn the value of listening over giving factual responses. Most encounters lasted 10–30 minutes, with responders mainly listening and validating feelings.

#### Challenge of exclusive focus on providing support.

One participant found it challenging to focus purely on support, as team members often expect her to provide solutions: *“Well, I am their manager, so they sort of expect me to come up with solutions. It was difficult to only focus on supporting them, it got a bit strange*.” However, she recognized the benefits of first addressing emotions through listening and comfort, then shifting to practical steps. This approach improved encounters and helped her develop skills to “*be there*” for her team.

#### Limited value of debrief form.

A debrief form was submitted for two of the encounters, which was less than the actual number of encounters. Participants reflected that it was not always easy to know when it counted as a formal encounter and that the task of debriefing took time and was not perceived to add value. *“I don’t know. I didn’t fill in the form after each encounter, some of them felt so short that I wasn’t sure if I was supposed to or not”.* As only two debrief forms were submitted, the data were limited and did not substantially inform the study’s conclusions; this low completion rate restricted our ability to draw quantitative insights from the debrief forms. Instead, our analysis relied primarily on focus group discussions and responder reflections, which provided valuable qualitative data to understand the implementation experience.

#### Importance of marketing.

*Researcher: “Have you shared information about RISE with the team?”*

*Responder: “Yes, we have… But everyone was not there… We should probably do it again. It is difficult, because we have just moved the clinic and everyone is busy”.*

Communicating RISE’s purpose was challenging due to competing demands to complete daily tasks. Responders emphasized the need having an active HWSP support team to remind staff about the program and assist responders in running it.

## Discussion

This pilot study suggests that the RISE program could be a suitable healthcare worker support program (HWSP) for veterinary practitioners. This aligns with recent findings that patient safety events are both common and psychologically impactful in veterinary settings, with nearly 80% of veterinarians reporting involvement in such events and a strong desire for peer-based support mechanisms [13]. Only minor adaptations were needed to tailor the program to veterinary practice, including cultural adjustments, decisions on responder compensation, evaluation methods, and debriefing formats. Additional suggestions included shortening training duration, incorporating veterinary-specific examples, and addressing financial conversations between clinicians and clients.

The RISE program was found to be acceptable and feasible for adaptation and implementation in veterinary settings, though further research is needed to confirm these findings in broader contexts. Both single-site and multi-site models were considered viable, each with distinct advantages and limitations.

To our knowledge, this is the first published report on the development and preliminary testing of an institutional healthcare worker peer-support program tailored for veterinary medicine. Given the increasing mental health challenges in the profession and projected staff shortages of up to 16% by 2030 [42], structured support systems like RISE could play a crucial role in improving staff retention, job satisfaction, and overall wellbeing.

Vetlife and Not One More Vet provide essential mental health support for veterinary professionals, mainly through helplines, crisis intervention, and peer networks. Unlike these broad-based initiatives, RISE offers structured, timely, institution-based peer support specifically after clinical incidents. Thus, RISE complements existing resources by addressing acute workplace distress within the organizational setting.

Following a clinical incident, many veterinary professionals may hesitate to seek support due to shame, fear of judgment, or uncertainty about how to access help. A structured HWSP like RISE may help mitigate these barriers by embedding support into the organizational culture, making it more visible, accessible, and confidential. While informal peer support is often present in small-animal practices, formalising these efforts may promote more consistent and equitable access across teams. Given the limited resources of many practices, full-scale implementation may not always be practical. However, integrating core principles, such as active listening, emotional validation, and psychological first aid, into continuing professional development is recommended in the literature as a cost-effective way to strengthen informal support networks and foster psychological safety [43].

This pilot study highlighted a tension between the professional expectation to “fix the problem” and the program’s primary goal of providing emotional support. This mirrors previous research showing that responders often struggle to balance problem-solving with emotional resilience [13,44,45]. It also raises the question of whether or not managers should serve as peer-responders. While this is debatable, the findings underscore the importance of equipping all clinical staff, including managers, with active listening skills.

Repeated communication about the program’s existence and purpose may help normalize its use, reduce stigma, and ensure accessibility. As Edrees and colleagues (2016) noted, sustained awareness through leadership engagement and regular reminders was critical to the early success of RISE [46]. Formal internal marketing is suggested to boost visibility; without it, even well-designed support systems may go underused, especially in high-stress settings like veterinary care.

Depending on organizational structure, either single-site or multi-site models can be adopted. A single-site setup offers localized support and responder consistency but may limit confidentiality and imposed a heavy workload on responders. A multi-site model enables broader access and distributed workload, fostering collaboration among responders. It requires technical coordination but facilitates usage tracking. These findings align with previous research in human healthcare [31,47].

Implementing a HWSP is complex, but most features of RISE are transferable to veterinary healthcare. Common barriers include unclear objectives and ownership [48], which were also noted by participants. Time constraints in busy practices challenged the length of the training phase, prompting suggestions to shorten it. However, role-playing and case examples were highly valued, and reducing these should be approached cautiously. Protecting time for clinicians and responders in high-stress departments, such as intensive care, may improve success [49]. In human healthcare, peer-responder roles have been integrated into existing positions to optimise resources [27]. Nomination systems for selecting responders have proven effective, yielding individuals already comfortable in the role [50]. Participants emphasized the importance of selecting responders with compassion and empathy, and noted that having a peer-responder leader or coach was beneficial [35]. Disseminating HWSP information is vital, as many workers are unaware of available support. Barriers include communication overload, language, and unclear messaging [51] Digital marketing boards and computer screen savers have been suggested to raise awareness [31].

Clear objectives and evaluation measures are essential but difficult to establish. In human healthcare, success is often gauged through participant perceptions rather than outcome metrics [52]. It would be worthwhile to evaluated program effectiveness using the second victim experience and support (SVEST) tool. Additionally, given the multinational composition of participants in this pilot, future research could benefit from cross-country or multi-clinic comparative studies to identify context-specific factors and enhance the generalizability of findings. This study also did not explore proactive versus reactive approaches to clinician support, which has been recommended for consideration when implementing a HWSP [32,50].

This study has several limitations. The RISE program was selected due to its widely established use, and other HWSPs may also be suitable. The use of snowball sampling and word-of-mouth recruitment may have introduced selection bias and limit the representativeness of our sample. The qualitative methodology reflects only participant perception of the program rather than actual experience, and emergent themes were generated by a single researcher, introducing potential bias. The study relied on subjective leader nomination of peer-responders, leading to a more highly skilled group than one based on volunteer candidates. Online training may have limited participant engagement and accuracy of the evaluation. The study’s focus on a pilot test with 48 participants over five months may not fully capture the long-term effectiveness or sustainability of the model and the limited scale and duration of the pilot indicate that the findings should be interpreted with caution and may not be generalizable to all contexts. Additionally, the lack of completed debrief forms after encounters further limited the ability to capture detailed data from responders. Additional limitations include the reliance on voluntary participation, which may have introduced self-selection bias; a predominantly European participant base, which may limit cultural generalizability; and a gender imbalance in leadership training respondents. However, the follow-up provided valuable insights, and the pilot offers a foundation for a broader-scale study.

In summary, this study provides initial, exploratory evidence that the RISE peer support program can be adapted and potentially effective for veterinary settings, but the limited scale and lack of formal outcome evaluation mean that further research is required to determine its effectiveness and generalizability. While full implementation has not yet been accomplished, the findings suggest that both single- and multi-site models are viable, each incorporating tailored adaptations. Further research using tools like the SVEST tool is recommended to evaluate effectiveness. In a profession facing increasing mental health challenges and workforce shortages [42,53], structured support programs like RISE may be a valuable strategy to enhance clinician wellbeing, staff retention [54] and care quality.

## Supporting information

S1 Appendix Peer-responder assessment debrief survey. (DOCX)
