# Supplementary material for: Peer support after clinical incidents in veterinary care: Adopting the RISE (Resilience In Stressful Events) program
Source: PLoS One. 2026 Jan 27;21(1):e0341324. doi: 10.1371/journal.pone.0341324 (PMC12843549; doi:10.1371/journal.pone.0341324)
Supplement: S1 Appendix — (DOCX) [file pone.0341324.s001.docx]

# Appendix 1

**Appendix 1. Peer Responder Assessment Debrief Survey**

*Initial call with clinician*

1. Did the clinician encounter any barriers to accessing RISE?
   1. Yes
   2. No

Comment

1. Were there any barriers to setting up a time/place to meet with the clinician?
   1. Yes
   2. No

Comment

1. Was this the first time you responded to a call from this person?
   1. Yes
   2. No

Comment

1. Had this person spoken with another RISE responder?
   1. Yes
   2. No

Comment

1. If she/he had spoken to another RISE provider, was it about the same event or a different event?
   1. Same event
   2. Different event

Comment

*Encounter with clinician*

1. After meeting with the person, did you feel you were able to provide helpful support?
   1. Yes
   2. No

Please comment:

1. As you listened to the person, did you find yourself wanting to “fix” the problem? If so, how did you respond to this feeling? (free text)
2. Was there a point during the discussion with the person when you struggled/felt uncomfortable? (free text)
3. What worked well during your encounter with the person? (free text)
4. Do you have worries or concerns that the person might have additional problems?
   1. Yes
   2. No
   3. If yes, what were they?
5. Did you experience any worries or concerns about how you responded? If so, please describe them.
   1. Yes
   2. No

Please comment:

1. Was there any information you would have liked to have prior to the encounter? If so, please describe. (free text)

*Training/education*

1. I felt comfortable listening to the person
   1. Strongly disagree
   2. Disagree
   3. Agree
   4. Strongly Agree
2. I felt comfortable responding to the person
   1. Strongly disagree
   2. Disagree
   3. Agree
   4. Strongly Agree
3. I need additional training/experience as a peer responder
   1. Strongly disagree
   2. Disagree
   3. Agree
   4. Strongly Agree
4. I am comfortable with my knowledge and skills as a peer responder
   1. Strongly disagree
   2. Disagree
   3. Agree
   4. Strongly Agree
5. I am highly competent as a peer responder
   1. Strongly disagree
   2. Disagree
   3. Agree
   4. Strongly Agree
6. I would be able to train other peer responders
   1. Strongly disagree
   2. Disagree
   3. Agree
   4. Strongly Agree
7. Which aspect of the Psychological First Aid (PFA) training you received was most beneficial to your role as a peer responder?
   1. Lecture presentation
   2. Role play
   3. RAPID acronym
   4. Video excerpts
   5. Handouts
   6. Narratives
   7. Other
8. Please rate your overall competence as a peer responder
   1. Poor
   2. Fair
   3. Good
   4. Very good
   5. Excellent

*Peer responder overall experience*

1. How would you rate your own emotional distress as a responder to this encounter (1 not at all painful – 10 extremely painful)?
   1. 1
   2. 2
   3. 3
   4. 4
   5. 5
   6. 6
   7. 7
   8. 8
   9. 9
   10. 10
2. I felt the need to refer the person to additional resources
   1. Strongly disagree
   2. Disagree
   3. Agree
   4. Strongly agree
3. I was able to offer the person additional helpful resources
   1. Strongly disagree
   2. Disagree
   3. Agree
   4. Strongly agree
4. I felt confident in offering the person additional resources
   1. Strongly disagree
   2. Disagree
   3. Agree
   4. Strongly agree
5. If you offered the person additional resources, what were they? (free text)

*Peer responder recommendations*

1. Overall how would you rate the success of this encounter?
   1. Poor
   2. Neutral
   3. Excellent
2. It was beneficial for the person to contact the RISE team:
   1. Strongly disagree
   2. Disagree
   3. Agree
   4. Strongly agree
3. I met the persons expectations:
   1. Strongly disagree
   2. Disagree
   3. Agree
   4. Strongly agree
4. I felt satisfied with how this encounter turned out:
   1. Strongly disagree
   2. Disagree
   3. Agree
   4. Strongly agree
5. This encounter suggests ways to improve the RISE program, including: (free text)
